# The Role of Habitat Complexity in Community Development Is Mediated by Resource Availability

**DOI:** 10.1371/journal.pone.0102920

**Published:** 2014-07-23

**Authors:** Rachel S. Smith, Emma L. Johnston, Graeme F. Clark

**Affiliations:** 1 Odum School of Ecology, University of Georgia, Athens, Georgia, United States of America; 2 Evolution and Ecology Research Centre, School of Biological, Earth and Environmental Science, University of New South Wales, Sydney, New South Wales, Australia; College of Charleston, United States of America

## Abstract

Habitat complexity strongly affects the structure and dynamics of ecological communities, with increased complexity often leading to greater species diversity and abundance. However, habitat complexity changes as communities develop, and some species alter their environment to themselves provide habitat for other species. Most experimental studies manipulate basal substrate complexity, and while the importance of complexity likely changes during community development, few studies have examined the temporal dynamics of this variable. We used two experiments to quantify the importance of basal substrate complexity to sessile marine invertebrate community development through space and time. First, we compared effects of substrate complexity at 70 sites across ten estuaries. Sites differed in recruitment and community development rates, and after three months provided spatial variation in community development stage. Second, we tested for effects of substrate complexity at multiple times at a single site. In both experiments, complexity affected marine sessile invertebrate community composition in the early stages of community development when resource availability was high. Effects of complexity diminished through time as the amount of available space (the primary limiting resource) declined. Our work suggests the presence of a bare-space threshold, at which structural complexity of the basal substrate is overwhelmed by secondary biotic complexity. This threshold will be met at different times depending on local recruitment and growth rates and is likely to vary with productivity gradients.

## Introduction

Habitat complexity, or the physical structure of an environment, influences community composition in a number of ways. Complex habitats can promote species coexistence by providing a wide range of niches, thereby reducing niche overlap and increasing diversity [1], [2]. Classic work by MacArthur & MacArthur (1961) found a correlation between bird species diversity and foliage height diversity, rather than plant species composition [3], and similar relationships between species diversity and habitat complexity have since been observed in terrestrial [4]–[6], freshwater [7]–[9], and marine systems [10], [11]. Habitat complexity can also be important in mediating predation, since cryptic habitats provide refuge for smaller organisms that would otherwise be vulnerable [12]–[15].

In many systems habitat complexity varies through space and time. Available structure can change seasonally [6], in response to disturbance [16], and as a result of interactions between species and their environment. Individual organisms can both reduce and add to habitat complexity: resource utilization decreases the amount of available substrate, but some species can themselves provide habitat for others. In marine communities, habitat-forming organisms such as barnacles and algae provide substrate for other organisms to settle and grow, and can become the main source of structure once basal substrate becomes rare [17], [18]. Effects of basal substrate complexity may therefore change over time, as the complexity of the substrate is buffered by habitat complexity provided by resident species.

Many studies have observed strong effects of basal substrate complexity on community structure, but few have examined how this changes over the course of community development. Southwood et al (1979) showed that the relative influence of habitat complexity changed over the course of succession in a birch woodland, and structural complexity became more important to species diversity in the later stages of succession [19]. A similar study by Brose (2003) in temporary wetlands suggested that structural complexity was independent of successional stage, and that the quantity of structural complexity determined community richness and diversity [20]. Work in fouling assemblages has suggested a declining importance of complexity effects with time, but has not explicitly compared stages of community development [21], [22].

In sessile invertebrate communities, population dynamics and community composition are dependent on space availability, as larvae require space to settle and grow [23]. Space is abundant in the early stages of community development, allowing active larval choice of settlement substrate [24]–[28]. Larvae may preferentially recruit to structural features of the environment, which can influence larval survival [29]. Fine-scale structural complexity changes the hydrodynamics, physical cues, and refuge quality of substrates, which may in turn alter larval settlement [25], [30]. Hydrodynamics may encourage settlement in grooves or crevices, as larvae become trapped in eddies that form on the leeward side of structural features [25]. Larvae may also choose to settle in the comparatively protected substrate of these features as a means of refuge from predators [31]–[33]. In hard-substrate environments, larvae frequently settle preferentially in structural features such as grooves, pits, or crevices [9], [32]–[34]. However, as the amount of available bare space declines during community development, the importance of basal substrate complexity to sessile invertebrate communities might also diminish and the role of biotic complexity may become more important.

Here we used two experiments to investigate how the role of habitat complexity changes throughout community development. We hypothesized that structural complexity would become less predictive of species abundance and diversity over time, as basal substrate is sequestered. Following previous studies, we represented habitat complexity by cutting varying numbers of grooves into the surface of a flat, hard substrate [32], [34], [35]. We considered variation in the stage of community development in two ways, through space and time. First, we compared communities across multiple estuaries, in which communities differed naturally in their assembly-rate and species composition. By comparing communities across estuaries at a single point in time, we captured communities at different stages of community development. Second, we observed recruitment density over the course of community development at multiple times. In each of these approaches we examined the relationship between basal substrate complexity and community composition. Together, these experiments offer insights into the generality of the relationships between habitat complexity and diversity in the context of community development for marine sessile invertebrate communities.

## Materials and Methods

### Ethics Statement

This study surveyed benthic invertebrate distributions across estuaries and experiments were conducted on invertebrate communities. Sampling in each location was approved and carried out in strict accordance with the New South Wales Department of Primary Industries and New South Wales Marine Parks (Permit No.P09/0072-1.0). Work at Chowder Bay, NSW was approved by the Sydney Institute of Marine Science, and no additional permits were needed for this site. All data collected in this work will be made available upon request.

We conducted two experiments to examine how the role of habitat complexity may change during community development. The first experiment utilized spatial variation in community development, and the second experiment utilized temporal variation.

### Complexity effects across space

#### Study sites and sampling design

**Figure 1:**
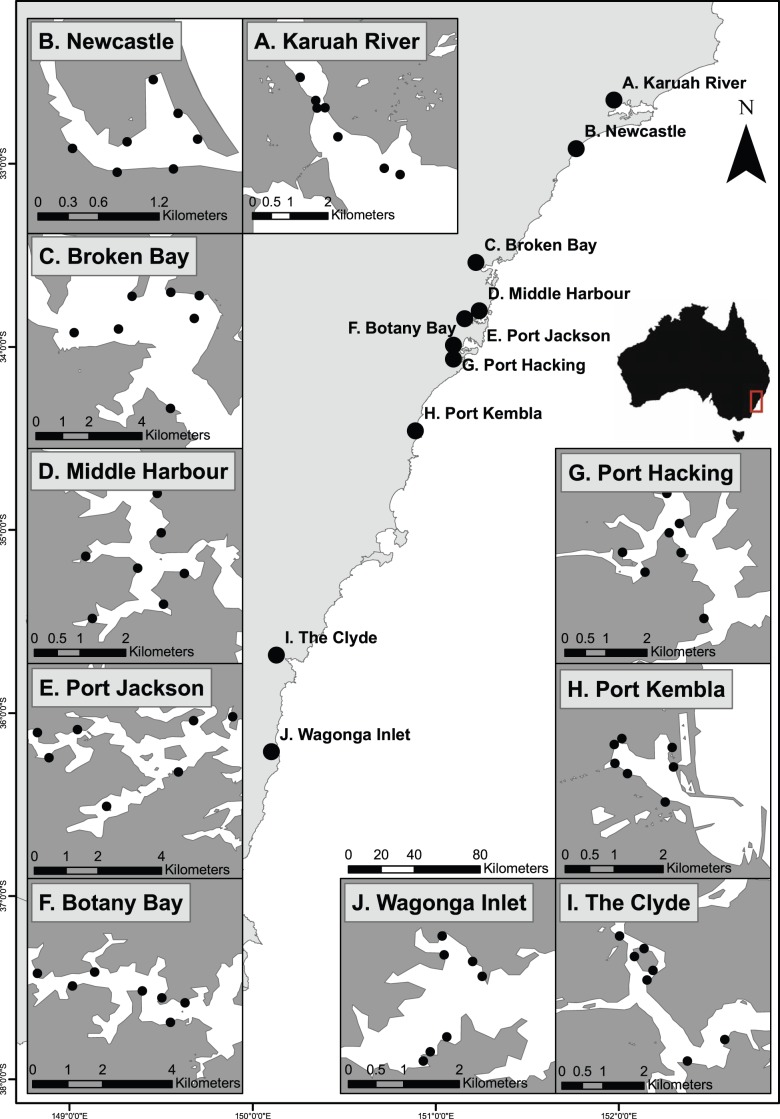
Study sites in New South Wales, Australia. Location of study sites for the spatial complexity experiment. Settlement plates were deployed for three months at seven sites located in each of ten focal estuaries along the southern coast of New South Wales, Australia.

This experiment was conducted at 70 sites across ten estuaries in New South Wales, Australia (Figure 1). All of these estuaries are permanently open tidal systems located within 500 km of each other along the coast. Seven sites were randomly chosen in each estuary for deployment of artificial substrata (hereafter “settlement plates”). In total, 560 settlement plates were cut from lightly sanded black Perspex (11×11×0.5 cm).

Various numbers of grooves (0, 4, 8 or 16) were cut into the plates to create four distinct levels of complexity. Evenly spaced grooves were cut across the full length of each plate to a depth of 0.5 mm, which approximated the size of settling larvae. One replicate of each treatment was attached to both sides of 60 cm×60 cm grey PVC panels using stainless steel fasteners and treatments were randomized on both sides of each panel. Panels were then attached to both a weight and a float and vertically deployed at 5 m depth at each site. Following deployment, the communities were left undisturbed to develop for three months (December 2010-March 2011).

#### Community census

After three months settlement plates were collected and communities were photographed and preserved in 7% formalin prior to weighing and census. Wet weight of settlement plates was noted prior to census as a measure of assemblage biomass. Percent cover of species was assessed with an 81-point count, whereby a grid was placed over the communities and the species present under each point were counted using a dissecting microscope. Each community was searched for species that were not observed in the point counts to determine total species richness per plate. Taxa were identified to the lowest possible taxonomic level.

#### Data Analysis

We analyzed effects of complexity on multivariate community structure across all ten estuaries using a three-factor PERMANOVA. Compositional differences between communities were quantified using Bray-Curtis similarities, a standard metric used to assess differences in multivariate community structure [36]. The main factor of interest was Complexity (fixed), and Estuary and Site (nested within Estuary) were random factors. Analyses showed that there was no significant interaction between complexity and site nested within estuary, so site was ignored as a nested factor in the analysis. Community data between estuaries were also compared with Principal Coordinate Analysis (PCO).

Analyses revealed considerable variation in the effects of complexity across estuaries that appeared to be correlated with the amount of bare space. To explore this correlation we first calculated the slope of relationships between complexity and biotic responses (e.g. species abundance or diversity) at each of the seven sites within each estuary. For each biotic response, we tested for a relationship between the slope of complexity effect at each site and amount of bare space. We did this by regressing the slope of the diversity-complexity relationship against the mean bare space at each site (n = 8). These tests were done with linear mixed models, using the ‘lme4’ package in R [37]. We conducted a separate PERMANOVA test for effects of complexity on community structure in Wagonga Inlet – the estuary with the most bare space. This test used the same PERMANOVA design as above, but using Complexity (fixed) and Site (random) as factors. Canonical analysis of principal coordinates for Wagonga Inlet was also performed to evaluate differences between complexity treatments. We also conducted a separate PERMANOVA test for effects of complexity on community structure in Port Jackson as an example of an estuary with high biotic cover.

### Complexity effects through time

#### Study sites and sampling design

A manipulative experiment was performed to examine the role of complexity at multiple times over the course of community development. This study was performed in Chowder Bay (33° 30′ 13″ S, 151° 9′ 10″ E), a protected inlet near the mouth of Sydney Harbor, New South Wales, Australia. Chowder Bay has a tidal range of 1 to 1.5 m and a jetty extends approximately 30 m from the shore into the bay. The jetty pilings support a diverse suite of subtidal sessile invertebrates, including solitary and colonial ascidians, bryozoans, sponges, polychaetes, and barnacles [38].

As in the spatial experiment, structural complexity was manipulated by cutting varying numbers of grooves (0, 4, 8 or 16) into the surface of 48 settlement plates. One replicate of each treatment was attached to each of six PVC panels, and treatments were randomized on each panel. Panels were hung vertically beneath the Chowder Bay pier at a depth of 1.5 meters below the low tide mark in December 2010. Half of the settlement plates were collected after one month for census, while the other half were left undisturbed for the full three months. Community composition was assessed at both one and three months to examine differences between initial colonization and long-term establishment, and to observe changes in recruitment through time.

#### Community Census

Plates were counted live, and were submerged in aerated seawater while awaiting census. Otherwise, census was conducted as described for the spatial complexity experiment, but using a 100-point grid.

#### Data Analysis

Multivariate community data were analyzed separately for communities collected after one and three months, using a one-factor PERMANOVA with Complexity as a fixed factor. Community data were then visualized with Principal Coordinate Analysis (PCO). Univariate analyses were subsequently performed for community diversity indices, species taxonomic groups, and for individual species that showed strong correlations with community differences in the Principal Coordinate Analysis (PCO). For the univariate analyses, residual plots were inspected to test for homogeneity of variance and data were log-transformed to improve homogeneity where necessary.

## Results

### Complexity effects across space

**Figure 2:**
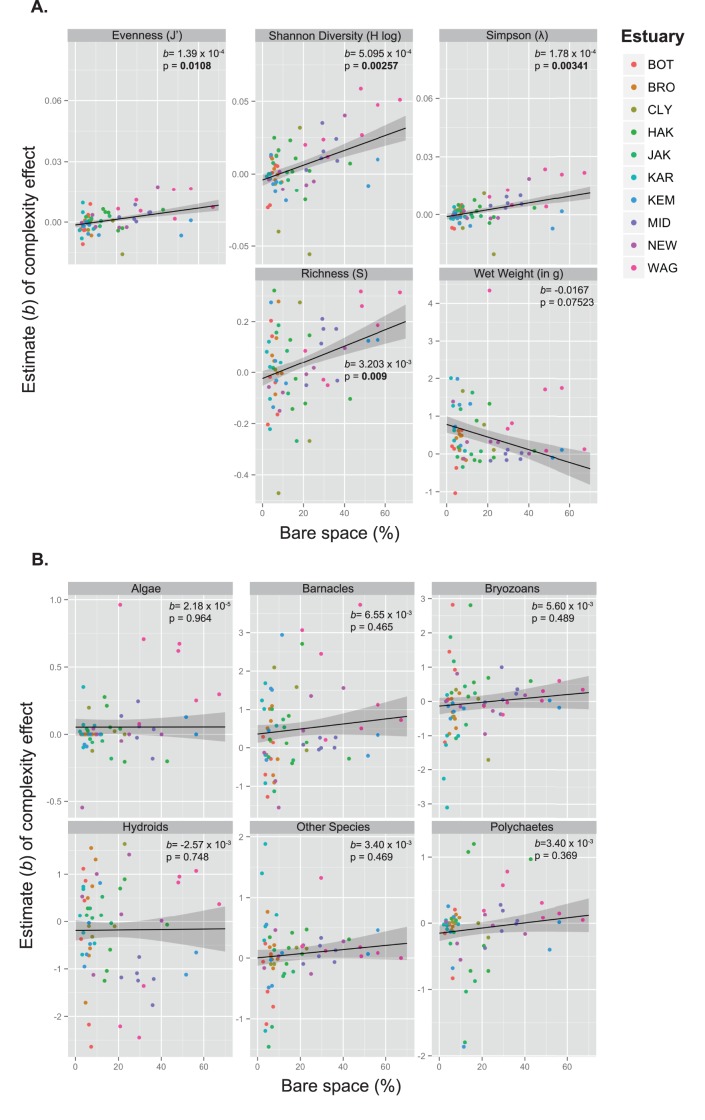
Relationships between the strength (slope) of the complexity effect at each site and the amount of bare space for the spatial complexity experiment. Lines are predicted values from linear mixed models, ± SE. (A) shows the response of diversity indices relative to bare space, and (B) shows the response of taxonomic groups. Areas shaded gray in the figure represent the standard error of the regressions, and slopes and p-values associated with diversity indices and species taxonomic groups are inset in each panel. Estuary labels correspond to Botany Bay (BOT), Broken Bay (BRO), the Clyde (CLY), Port Hacking (HAK), Port Jackson (JAK), Karuah River (KAR), Port Kembla (KEM), Middle Harbour (MID), Newcastle (NEW), and Wagonga Inlet (WAG).

**Figure 3:**
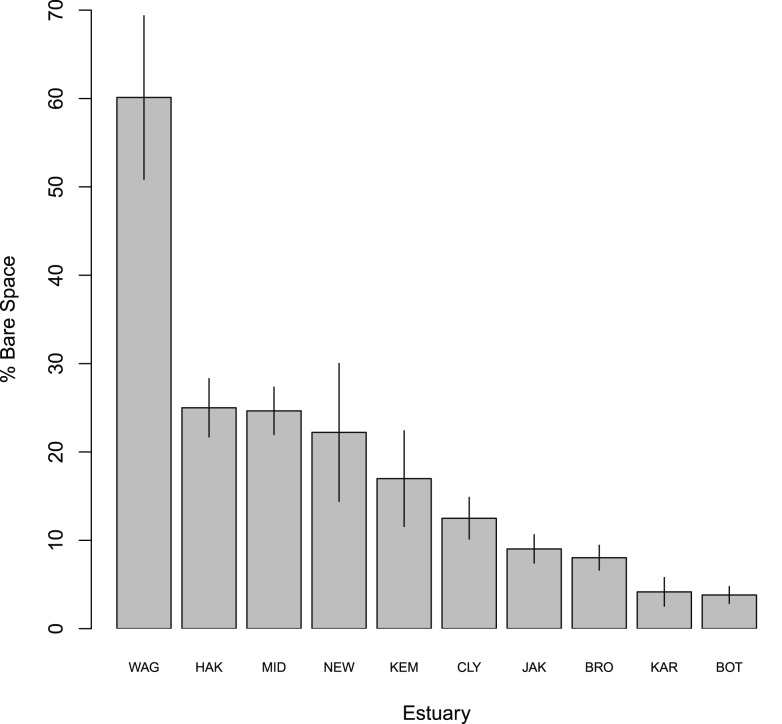
Mean (± SE) percent cover of bare space for control complexity treatments in each estuary for the spatial complexity experiment. Control complexity treatments refer to those settlement plates without any added basal complexity, no grooves. Estuary label abbreviations follow those in Figure 2, and estuaries are organized by decreasing percent bare space.

Complexity treatments led to differences in the structure of communities, but effects varied among estuaries (PERMANOVA, Treatment x Estuary, Psuedo-*F*_27,166_ = 1.384, *P*<0.001; Table 1). Principal Coordinate Analysis (PCO) showed large differences between estuaries, but did not offer further insight into general complexity effects (Figure S1). Regression analysis revealed that effects of complexity on community diversity, wet weight, and individual species abundances were strongest in estuaries with the greatest bare space (Figure 2). Linear mixed models showed that the effect (slope) of complexity on several diversity indices was proportional to the amount of bare space available (Figure 2A). In fact, some sites with high biotic cover exhibited declines in diversity parameters. Similar effects were observed for percent cover of some individual species groups (Figure 2B). Complexity effects were strongest in Wagonga Inlet where average bare space was over 50%, and weaker where there was less bare space (Figure 3).

**Table 1:** Permutational multivariate analysis of variance (PERMANOVA) testing differences between complexity treatments (0, 4, 8, 16) across estuaries for the spatial complexity experiment.

Source	df	MS	Pseudo-F	p-value
Complexity	3	1575	1.6211	0.0304
Estuary	9	32336	8.7933	0.0001
Site (Estuary)	59	3835.1	5.3845	0.0001
Complexity x Estuary	27	977.31	1.3842	**0.0002**
Complexity x Site (Estuary)	163	706	0.99122	0.5737
Residuals	246	712.26		
Total	507			

Significant p-values (<0.05) involving fixed factors (i.e., Complexity) are in bold. Bray-Curtis similarities were used to quantify multivariate community structure.

**Figure 4:**
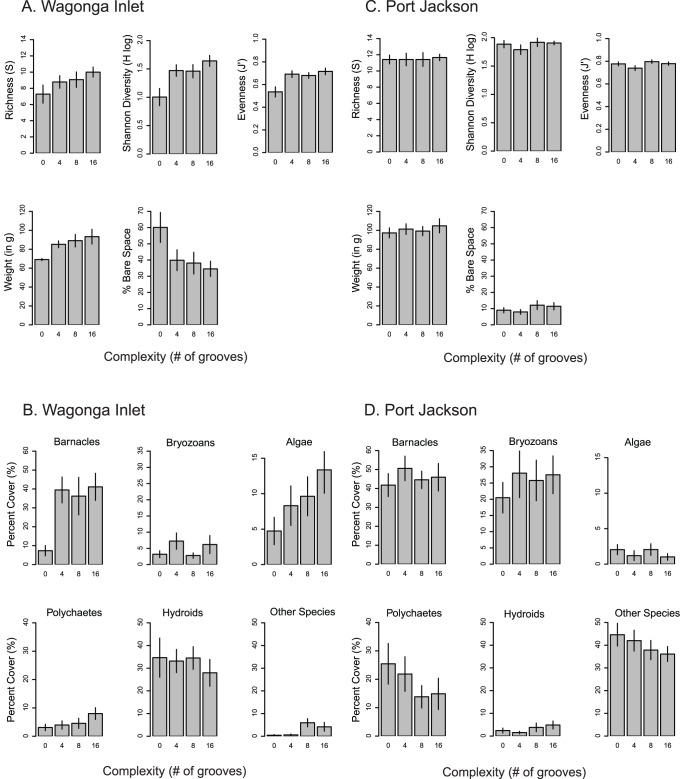
Diversity indices and abundance of taxonomic groups in complexity treatments from Wagonga Inlet (A, B) and Port Jackson (C,D), demonstrating high and low percentages of bare space respectively. Bars represent means of replicate plates, ± SE. Complexity refers to the number of grooves cut into the surface of settlement panels (0, 4, 8, or 16).

We analyzed data from Wagonga Inlet separately to examine the nature of complexity effects. Multivariate analysis indicated strong effects of complexity on community structure within this estuary (PERMANOVA, Pseudo-*F*_3,18_ = 3.456, *P*<0.001; Table 2). Species numbers, richness, diversity, and biomass all increased in the presence of complexity, and there was a corresponding decrease in bare space (Figure 4A). Some major taxonomic groups were also more abundant in high complexity treatments, particularly barnacles, algae and polychaetes (Figure 4B). Taxonomic groups responded differently to varying degrees of complexity. For example, barnacles responded to the mere presence of complexity, and increasing structural complexity did not alter the barnacle complexity response. Other species groups, such as algae and polychaetes, exhibited linear responses to complexity, while some groups, such as hydroids, did not show differences in abundance with increasing complexity. Canonical analysis of principal coordinates did not show distinct patterns with regard to the complexity effect, limiting our interpretation of whether complexity effects were dependent on species identity (Figure S2).

**Table 2:** Permutational multivariate analysis of variance (PERMANOVA) testing differences between complexity treatments (0, 4, 8, 16) within Wagonga Inlet and Port Jackson for the spatial complexity experiment.

Wagonga Inlet				
Source	df	MS	Pseudo-F	p-value
Complexity	3	2470	3.4568	**0.001**
Site	6	3446.7	4.3562	0.001
Complexity x Site	18	714.53	0.90307	0.717
Residuals	28	791.23		
Total	55			
**Port Jackson**				
**Source**	**df**	**MS**	**Pseudo-F**	**p-value**
Complexity	3	538.01	0.80948	0.703
Site	8	3927.3	6.6693	0.001
Complexity x Site	18	668.06	1.1345	0.25
Residuals	24	588.86		
Total	53			

Significant p-values (<0.05) involving fixed factors (i.e., Complexity) are in bold. Bray-Curtis similarities were used to quantify multivariate community structure.

We performed the same analysis for community data collected from Port Jackson as an example of the patterns of community diversity and species abundance observed in estuaries with high biotic cover and minimal available bare space (Figure 4). Multivariate analysis did not show significant effects of complexity on community structure within this estuary (PERMANOVA, Pseudo-F_3,18_ = 0.80948, P = 0.703; Table 2). For Port Jackson, there were no consistent patterns for any of the diversity indices (Figure 4C) or individual species groups (Figure 4D) with increasing basal complexity. As in Port Jackson, other estuaries with high biotic cover showed similar irrelevance of basal complexity to species diversity and abundance. Canonical analysis of principal coordinates was not performed for Port Jackson, as significant complexity effects were not observed.

### Complexity effects through time

**Figure 5:**
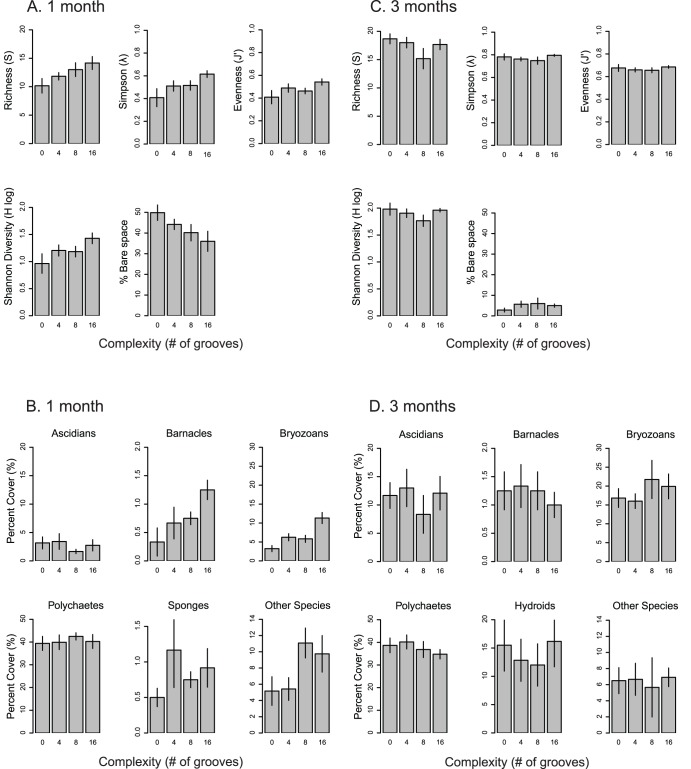
Diversity indices and abundance of taxonomic groups in complexity treatments after one month (A, B) and after three months (C, D), demonstrating high and low percentages of bare space respectively. Bars represent means of replicate plates, ± SE. Complexity refers to the number of grooves cut into the surface of settlement panels (0, 4, 8, or 16). No hydroids were observed after one month, but hydroids were abundant after three months.

PERMANOVA found a significant difference in communities between complexity treatments after one month (Pseudo-*F*_3,20_ = 1.751, *P* = 0.015; Table 3) but not after three months (Pseudo-*F*_3,20_ = 0.592, *P* = 0.978; Table 3). After one month, species number, richness, and diversity were greater in treatments with more complexity, and there was a corresponding decline in bare space with increasing complexity (Figure 5A). Similarly, several major taxonomic groups showed greater abundances in higher complexity treatments after one month, including barnacles, bryozoans, and other species (Figure 5B). However, these patterns were not observed after three months, and no strong patterns were observed for either species diversity indices (Figure 5C) or major taxonomic groups (Figure 5D) at this later time point. Overall, abundances of ascidians, bryozoans, and hydroids increased significantly with community development from one month to three months. Principal Coordinate Analysis (PCO) did not show distinct patterns with regard to complexity effects after either one month or three months, limiting our interpretation of whether complexity effects were dependent on species identity (Figure S3, Figure S4).

**Table 3:** Permutational multivariate analysis of variance (PERMANOVA) testing differences between complexity treatments (0, 4, 8, 16) after one and three months for the temporal complexity experiment.

1 month				
Source	df	MS	Pseudo-F	p-value
Complexity	3	989.69	1.7512	**0.0375**
Residuals	20	565.17		
Total	23			
**3 month**				
**Source**	**df**	**MS**	**Pseudo-F**	**p-value**
Complexity	3	423.2	0.59219	0.944
Residuals	20	714.64		
Total	23			

Significant p-values (<0.05) are in bold. Bray-Curtis similarities were used to quantify multivariate community structure.

**Figure 6:**
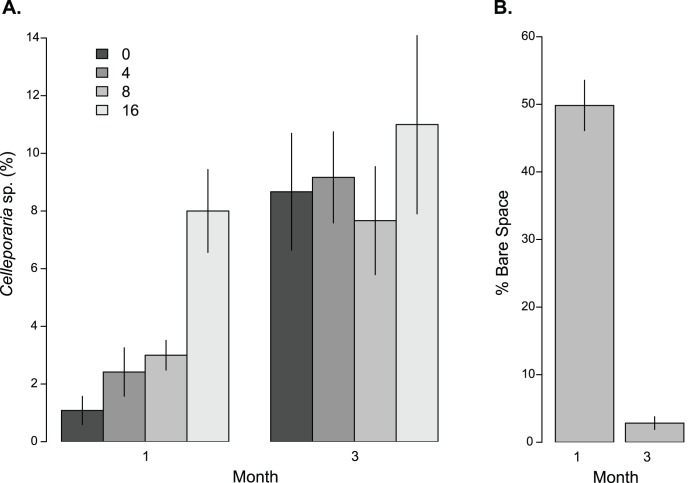
Mean (± SE) percent cover of (A) *Celleporaria sp.* and (B) bare space for control complexity treatments, after one and three months in the temporal complexity experiment. Color gradations correspond to complexity treatment and refer to the number of grooves cut into the surface of settlement panels (0, 4, 8, or 16).

We then performed univariate analyses for community diversity indices and species taxonomic groups (Table 4) to illuminate the driving factors of complexity effects. Of these factors, bryozoans showed the strongest response to increasing diversity, and these patterns were driven primarily by one species, the bryozoan *Cellporaria* sp. As for the multivariate community analysis, cover of *Celleporaria* sp. increased with complexity after one month (*F*_3,20_ = 11.16, *P*<0.001; Table 4), but no difference was detectable after three months (F_3,20_ = 0.396, P = 0.757; Fig. 6A; Table 4). No significant differences were observed for any diversity indices, species taxonomic groups, or individual species after three months (Table 4). Mean bare space on control plates declined over time (Figure 6), suggesting that complexity effects only occur when there is a substantial amount of bare space.

**Table 4:** Univariate analysis of diversity indices, taxonomic species groups, and individual focal species testing differences between complexity treatments (0, 4, 8, 16) after one and three months for the temporal complexity experiment.

1 month				
Source	df	MS	F	p-value
Richness (S)	3	17.486	2.42	0.0961
Residuals	20	7.225		
Total Individuals (N)	3	239.36	2.663	0.0758
Residuals	20	89.89		
Evenness (J’)	3	0.018471	2.021	0.143
Residuals	20	0.009139		
Shannon Diversity (H log)	3	0.2170	2.341	0.104
Residuals	20	0.0927		
Simpson (λ)	3	0.04291	2.628	0.0784
Residuals	20	0.01633		
Ascidians	3	3.583	0.577	0.637
Residuals	20	6.212		
Barnacles	3	0.8611	3.179	**0.0463**
Residuals	20	0.2708		
Bryozoans	3	68.64	10.14	**0.00286**
Residuals	20	6.77		
Polychaetes	3	11.15	0.235	0.871
Residuals	20	47.49		
Sponges	3	0.4722	0.827	0.494
Residuals	20	0.5708		
Other Species	3	54.37	2.681	0.0745
Residuals	3	20.28		
*Botrylloides* sp.	3	0.1215	0.198	0.897
Residuals	20	0.6146		
*Smittinidae* sp.	3	0.5972	0.843	0.486
Residuals	20	0.7083		
*Diplosoma* sp.	3	5.927	1.163	0.349
Residuals	20	5.098		
*Schizoporella* sp.	3	1.0382	1.678	0.204
Residuals	20	0.6188		
*Celleporaria* sp.	3	54.90	11.16	**0.000161**
Residuals	20	4.92		
**3 month**				
**Source**	**df**	**MS**	**Pseudo-F**	**p-value**
Ascidians	3	24.87	0.46	0.713
Residuals	20	54.02		
Barnacles	3	0.1250	0.199	0.896
Residuals	20	0.6292		
Bryozoans	3	43.07	0.62	0.61
Residuals	20	69.47		
Polychaetes	3	32.87	0.596	0.625
Residuals	20	55.19		
Hydroids	3	24.49	0.237	0.87
Residuals	20	103.36		
Other Species	3	1.76	0.055	0.983
Residuals	20	32.09		
*Celleporaria* sp.	3	11.71	0.396	0.757
Residuals	20	29.57		

Significant P-values (<0.05) are in bold.

## Discussion

Basal habitat complexity was important to marine sessile invertebrate community composition, but only in the early stages of community development when resource availability was high. The rate of space sequestration differed between and within estuaries and thus the effects of basal habitat complexity varied through space. Effects were strongest in Wagonga Inlet where recruitment and growth were low and there was the most available space. Diversity indices and species abundances increased with fine-scale complexity in this estuary, but effects were weaker elsewhere. Basal complexity effects also appeared to diminish with time. Complexity effects were present after one month when there was a significant amount of bare space, but not after three months when space availability was low. Both the spatial and temporal studies suggest that bare space, or perhaps the absence of biotic habitat complexity, may mediate the role of basal complexity in community development of sessile marine invertebrate communities. However, these results may also apply more generally to other environments, such as bare soil and freshwater rocks, in which bare substrate is actively colonized [39], [40].

In our study, the mere presence of basal complexity in space-rich environments increased species diversity and abundance. Increasing complexity generally did not affect community patterns beyond the changes observed at the lowest level of complexity, indicating a threshold beyond which the effect of complexity remains constant. There is clearly a minimum level at which species begin to respond to complexity, which has been documented in fish and invertebrates as function of predation refuge [12], [41]. Species response to complexity is likely a matter of scale, and Kelaher (2003) suggests that there is an upper threshold at which the addition of more structural components leads to a decline in species diversity and abundance [42]. Our results suggest that our complexity treatments provided a level of complexity intermediate to these two potential thresholds. The role of complexity is likely also dependent on whether species exhibit mobile or sessile life histories [34]. For example, the importance of complexity as a predation refuge may be more important to sessile species in the vulnerable early stages of development, while mobile species may continue to find refuge in habitat complexity over the course of their lifetimes. However, regardless of mobility, the importance of complexity is likely dependent on the scale of complexity relative to species size.

Our work suggests the existence of a bare-space threshold at which the presence of structural complexity of the basal substrate becomes irrelevant to community development. Effects of complexity diminished rapidly when the availability of bare space fell below 30 to 50% in both the spatial and temporal experiments, although additional time points may be necessary in future studies to more clearly define the boundaries of a temporal threshold. When bare space is available, settling organisms can take advantage of fine-scale structural features of the basal substrate. However, as bare space becomes increasingly limited over the course of community development, or as a result of other factors such as high larval supply or settlement rate, the subtleties of fine-scale structure may be overwhelmed by biotic structure from recruitment.

The presence of primary recruits alters both larval settlement rates and post-settlement mortality by controlling available space [17], [18] and creating new micro-habitats for subsequent recruits [43]. Further, chemical cues may induce larvae to settle on resident adults rather than other structures [44], [45]. There may be a certain recruit density at which resident species facilitate future settlement to a greater extent than the presence of basal structural features. In fact, in our spatial complexity experiment, we even observed declines in species richness and abundance at sites with high biotic cover and minimal bare space – a relationship that may depend on the identity of primary recruits. For example, initial settlement of complex basal substrate by a dominant or competitive species could lead to declines in the richness and abundance of future settlers via competitive exclusion.

It is difficult to determine the role of species identity in our observed community patterns. In our spatial study, large differences in community composition among estuaries make it hard to isolate the role of species identity in development from complexity effects. However, broadly speaking, in communities with distinct complexity effects (e.g. Wagonga Inlet in the spatial study or after one month in the temporal study), barnacle and bryozoan taxonomic groups were significant drivers of complexity patterns, despite the fact that these species have different life history strategies (i.e. solitary vs. colonial). Colonial organisms are expected to dominate solitary species over the course of community development, and may be superior competitors in space-limited environments as a result of asexual, indeterminate growth and fouling ability [46]. Further work in this system should address the importance of species identity, specifically with regard to coloniality, in mediating fine-scale basal complexity effects. However, the fact that we did not see differences based on species identity in our study strengthens the suggestion that an alternative mechanism, such as bare space availability, may be more important in this system.

In our study, early differences in recruitment patterns, as driven by the presence of structural complexity, did not translate to long-term differences in the mature communities. This result has implications for understanding the importance of priority effects in marine benthic communities [47]. Sutherland and Karlson (1977) showed that after the initial developmental period in a sessile invertebrate community, subsequent changes to the community were dependent on the identity of both the resident adults and the newly settling larvae, and thus on the order of recruitment by distinct species. In long-term populations, adult mortality led to the release of approximately 20–60% of bare space annually, and the colonization of this space by new species dramatically changed community composition over time [48]. The fact that our communities became increasingly similar to each other over the course of community development suggests that priority effects become less pertinent with time, barring space-freeing processes such as adult mortality, disturbance, or predation that allow for variable larval settlement. Accordingly, more recent work has shown that interactions between resident adults and new recruits that affect juvenile persistence are strongest within hours of larval settlement [49].

The relationship between complexity effects and bare space availability suggests that the presence of basal habitat structure may be more important in communities that are recruitment limited. Recruitment limitation is the idea that population size and species densities may be limited by larval supply [50], [51]. “Supply-side” processes such as larval supply, settlement rate, and post-settlement mortality are closely tied to the presence of bare space. Roughgarden, Iwasa, and Baxter (1985) explore the role of bare space and “supply-side” factors in their classic model for the demography and population dynamics of an open population with space-limited recruitment. They suggest that in the presence of a low settlement rate, a steady state is reached where free space is present and the relative spatial abundance of species is determined by variation in settlement and mortality rates [52]. Thus, in recruitment-limited environments, the presence of ample bare space may support variable community responses to available structural features.

Previous work in marine and terrestrial systems have shown largely positive effects of complexity on species diversity measures, but few studies have defined the conditions in which this is important. This study suggests that fine-scale habitat complexity increases marine sessile invertebrate diversity measures and species abundance, but only in the early stages of community development. There may be a bare-space threshold at which structural complexity becomes overwhelmed by recruitment and community development loses sensitivity to structural complexity. However, this threshold will be met at different times depending on local recruitment and growth rates and is therefore likely to vary with gradients of productivity.

## Supporting Information

Figure S1 **Unconstrained Principal Coordinate Analysis (PCO) plot of community composition across estuaries by complexity treatment for spatial complexity experiment.** Vectors indicate the most important species driving sample spread (Pearson Correlation >0.6).
(EPS)

Figure S2 **Canonical analysis of principal coordinates (CAP) of community composition within Wagonga Inlet by complexity treatment for spatial complexity experiment.** Vectors indicate the most important species driving sample spread (Pearson Correlation >0.6).
(EPS)

Figure S3 **Unconstrained Principal Coordinate Analysis (PCO) plot of community composition after one month by complexity treatment for temporal complexity experiment.** Vectors indicate the most important species driving sample spread (Pearson Correlation >0.6).
(EPS)

Figure S4 **Unconstrained Principal Coordinate Analysis (PCO) plot of community composition after three months by complexity treatment for temporal complexity experiment.** Vectors indicate the most important species driving sample spread (Pearson Correlation >0.4).
(EPS)
